# Investigating dynamic structural and mechanical changes of neuroblastoma cells associated with glutamate-mediated neurodegeneration

**DOI:** 10.1038/srep07074

**Published:** 2014-11-17

**Authors:** Yuqiang Fang, Catherine Y. Y. Iu, Cathy N. P. Lui, Yukai Zou, Carmen K. M. Fung, Hung Wing Li, Ning Xi, Ken K. L. Yung, King W. C. Lai

**Affiliations:** 1 Department of Mechanical and Biomedical Engineering, City University of Hong Kong, Kowloon, Hong Kong; 2 Department of Biology, Hong Kong Baptist University, Kowloon, Hong Kong; 3 Hong Kong Productivity Council, Tat Chee Avenue, Hong Kong; 4 Department of Chemistry, Hong Kong Baptist University, Kowloon, Hong Kong; 5 Michigan State University, East Lansing, USA

## Abstract

Glutamate-mediated neurodegeneration resulting from excessive activation of glutamate receptors is recognized as one of the major causes of various neurological disorders such as Alzheimer's and Huntington's diseases. However, the underlying mechanisms in the neurodegenerative process remain unidentified. Here, we investigate the real-time dynamic structural and mechanical changes associated with the neurodegeneration induced by the activation of N-methyl-D-aspartate (NMDA) receptors (a subtype of glutamate receptors) at the nanoscale. Atomic force microscopy (AFM) is employed to measure the three-dimensional (3-D) topography and mechanical properties of live SH-SY5Y cells under stimulus of NMDA receptors. A significant increase in surface roughness and stiffness of the cell is observed after NMDA treatment, which indicates the time-dependent neuronal cell behavior under NMDA-mediated neurodegeneration. The present AFM based study further advance our understanding of the neurodegenerative process to elucidate the pathways and mechanisms that govern NMDA induced neurodegeneration, so as to facilitate the development of novel therapeutic strategies for neurodegenerative diseases.

Neurodegeneration mediated by glutamate receptors leading to cognitive deficit and dementia has been extensively studied^1^. Glutamate receptors are channels across cytomembrane that facilitates excitatory neurotransmission in the mammalian central nervous system (CNS)^2^,^3^. As a subtype of glutamate receptors, N-methyl-D-aspartate (NMDA) receptors take main function of synaptic plasticity such as long-term potentiation (LTP) and long-term depression (LTD) which is crucial for learning and memory^4^,^5^,^6^. However, the excessive stimulation of NMDA receptors results in different pathological conditions in neurodegenerative disorders such as stroke, ischemia and trauma^7^,^8^. Particularly, the ionotropic NMDA receptor is found to be both ligand-gated and voltage-dependent^9^ and the activation of NMDA receptors allows the extracellular Na^+^ and Ca^2+^ to flow into cell and intracellular K^+^ to flow out of cell^10^. Accumulating evidence indicated that the NMDA receptors facilitated Ca^2+^ influx into neurons, and thus mediated the array of neurodegenerative process in acute insults such as epileptic seizures, stroke and trauma^11^, and chronic diseases such as Huntington disease^12^ and Alzheimer's disease^13^. Therefore, the NMDA receptor played a significant role in inducing the pathogenesis of neurodegenerative diseases at the early stage.

Several possible mechanisms and pathways for the development of NMDA receptors-neurodegenerative diseases have been reported recently. These reports indicated that NMDA receptors-medicated neuronal plasticity or degeneration is mainly developed through the pathway, involving regulating dynamics of actin filaments and modulating a large group of intracellular Rho GTPase and actin-binding proteins. Rho GTPases, including Rac, Cdc42 and Rho, are main regulators in neuroplasticity by reorganizing actin filaments^14^. Rac and Cdc42 mainly contribute to the growth of actin filaments by leading *de novo* actin polymerization to form bundles of long actin filaments (filopodia), and branching short actin polymerization to form actin filaments network (lamlipodia)^15^. In addition, the major function of RhoA, a dominant sub-member of Rho, is to induce neurite retraction and cell rounding mainly through activation of motor proteins such as myosin II, and to generate contractile force among actin filaments^16^. By regulation of Rho GTPases, neuron conducts alternate action of growing and retracting and further facilitates axon extension, guidance and branching, and dendritic morphogenesis^14^. In many neurodegenerative disorders, the neuron exhibits atrophic processes which are caused by the misregulation of neuronal contraction. Specifically, an NMDA receptor-associated Rho GTPase-activating protein, p250GAP, was found to regulate dendritic spine morphological plasticity by modulating RhoA, which was related to the development of cognitive deficits in humans^17^. Moreover, the excessive activation of NMDA receptors and the subsequent Ca^2+^ influx rapidly activated RhoA and p38α that results in neuronal excitotoxicity^18^. Besides, a great number of actin-binding proteins such as α-actinin-2^19^, gelsolin^20^ and microtubule-associated protein 2 (MAP2)^21^ were identified as calcium/calmodulin regulated proteins. Based on all these studies, monitoring the morphogenesis and dynamics of cytoskeleton are crucial to further elucidating the pathways and identifying the detailed mechanism in the NMDA receptors-induced neurodegenerative process. Most of the reported studies focused on the subsequent morphogenesis of neurites (e.g. dendritic spine and growth cone) under activation of NMDA receptors^22^,^23^,^24^. However, little is known about the dynamic behavior of neuronal cells under stimulus of NMDA receptors, particularly about the reorganization of actin filaments during the NMDA receptors-induced neurodegeneration.

Actin filaments are one of the major components of cytoskeleton^25^. The actin filaments take major contribution to cytoskeleton and mainly spread out in cellular cortex^26^. As the main structural framework, actin filaments closely interact with receptors in cytomembrane (e.g., NMDA receptors), and maintain the cellular integrity by carrying tensile and compressive forces^27^. Therefore, the mechanical properties of cells (such as elasticity and surface roughness) are directly linked to the dynamics of actin filaments. Several measurement techniques have been developed for studies of cell mechanics^28^ such as micropipette aspiration^29^, magnetic tweezers^30^, optical tweezers^31^ and nanoindentation based on AFM^32^,^33^,^34^. Among all these techniques, AFM takes advantage in providing high-resolution 3D images of cellular topography at the molecular scale, as well as preforming real-time and quantitative elasticity measurement of live cells in physiological-like conditions^35^,^36^,^37^,^38^.

NMDA receptors induced changes in morphology of rat neurons was first demonstrated by using AFM, which was suggested to be caused by depolymerization of actin filaments^39^. More recently, AFM measurements showed that activation of NMDA receptors in cortical neurons caused abrupt increasing in modulus of elasticity due to the elevated hydrostatic pressure inside neurons^40^. However, the dynamic mechanical response of neurons during the neurogenative process is still not fully revealed. Here, we report, for the first time, the time-dependent behavior of both the mechanical properties and morphology of neuroblastoma cells under stimulus of NMDA receptors. The present AFM approach for monitoring the real-time changes of cell structure, surface roughness and stiffness allows us to obtain more detailed information on the NMDA associated degenerative process of live neuronal cells, and also opens up a promising domain for the anti-neurodegeneration drug screening.

## Results

### Studies of neuronal morphogenesis under stimulation of NMDA receptors by AFM imaging and surface roughness analysis

**Figure 1:**
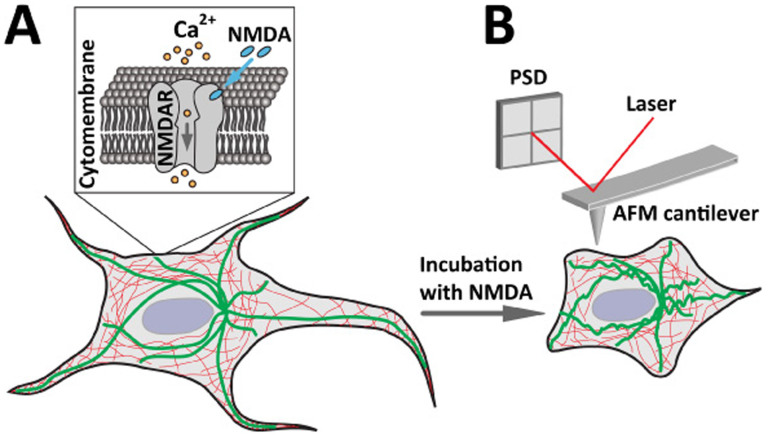
Schematic diagrams showing the NMDA induced structural changes of live cell that can be detected by AFM. (A) The process of the cell response under NMDA treatment. NMDA specifically binds to the NMDA receptors (NMDAR) in the cytomembrane that opens the ligand gated ion channel to facilitate Ca^2+^ influx into the cell. (B) AFM measurement of live cell response under NMDA treatment. NMDA treatment and Ca^2+^ influx induce reorganization of cytoskeleton (thick and green lines indicate microtubules, thin and red lines indicate actin filaments) and the resulting structural changes of the cell can be detected by AFM in high resolution and real time.

NMDA receptors were known to facilitate neuronal plastic changes in the brain^13^, in which the reorganization of cytoskeletal components is prominent either in normal synaptic plasticity mediated learning processes^41^ or in excessive stimulus of NMDA receptors induced neurodegenerative processes^42^. We hypothesized that when the NMDA specifically bind to NMDA receptors, the ligand gated ion channel is opened that causes Ca^2+^ influx (Fig. 1A). The excessive Ca^2+^ influx induces the reorganization of cytoskeleton, resulting in the structural change of the cell that could be detected by AFM in real time (Fig. 1B). Morphological changes of neuronal cells caused by disruption of the actin filaments was reported previously^43^, and thus in the present study, AFM imaging was conducted to reveal the morphology of live neuronal cells under different conditions. In the experiment, the AFM tip scanned across the selected neuronal cell surface line-by-line and the corresponding topographical image of the cell was generated.

SH-SY5Y cell line was used in the present study because it is a human-derived neuroblastoma cell with neuronal function and differentiation and is widely used as in vitro model for neuronal studies. It was reported that SH-SY5Y cells have the major typical features of normal neurons including dopaminergic character^44^ and expression of NMDA receptors^45^. SH-SY5Y cell treated by neurotoxin, such as β-amyloid^46^ and α-synuclein^47^, was widely investigated by using optical microscopy to evaluate the toxic effects of the neurotoxin, and further linking the response of the SH-SY5Y cell to the in vitro neuronal model of human diseases such as Alzheimer's and Parkinson's disease. Particularly, SH-SY5Y cell under activation of NMDA receptors was morphologically and fluorescently analyzed to indicate the neurodegeneration with the aim to develop the therapeutic strategy for the NMDA receptors induced neurodegeneration^48^,^49^.

**Figure 2:**
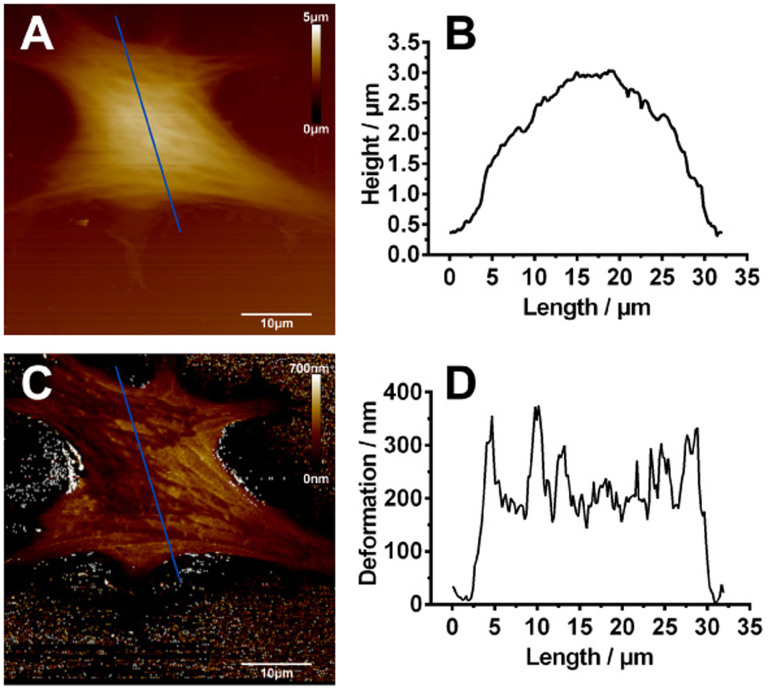
Representative AFM images of normal SH-SY5Y cells. (A) AFM height image and (B) height profile across the section line as marked in (A). The height of the cell is approximately 3 μm. (C) AFM deformation image and (D) deformation profile across the section line as marked in (C). The average deformation of the cell is about 250 nm. (scale bar: 10 µm).

A representative AFM height image of live SH-SY5Y neuronal cells was obtained (Fig. 2A) and the corresponding cross-sectional height profile indicates that the height of the cell was approximately 3 μm (Fig. 2B). Since the cell was relatively soft, the cell membrane was deformed by the AFM tip during the scanning and the deformation information of the entire cell was obtained from the deformation image and the cross-sectional deformation profile (Fig. 2C, D). The average deformation of the cell was about 250 nm.

**Figure 3:**
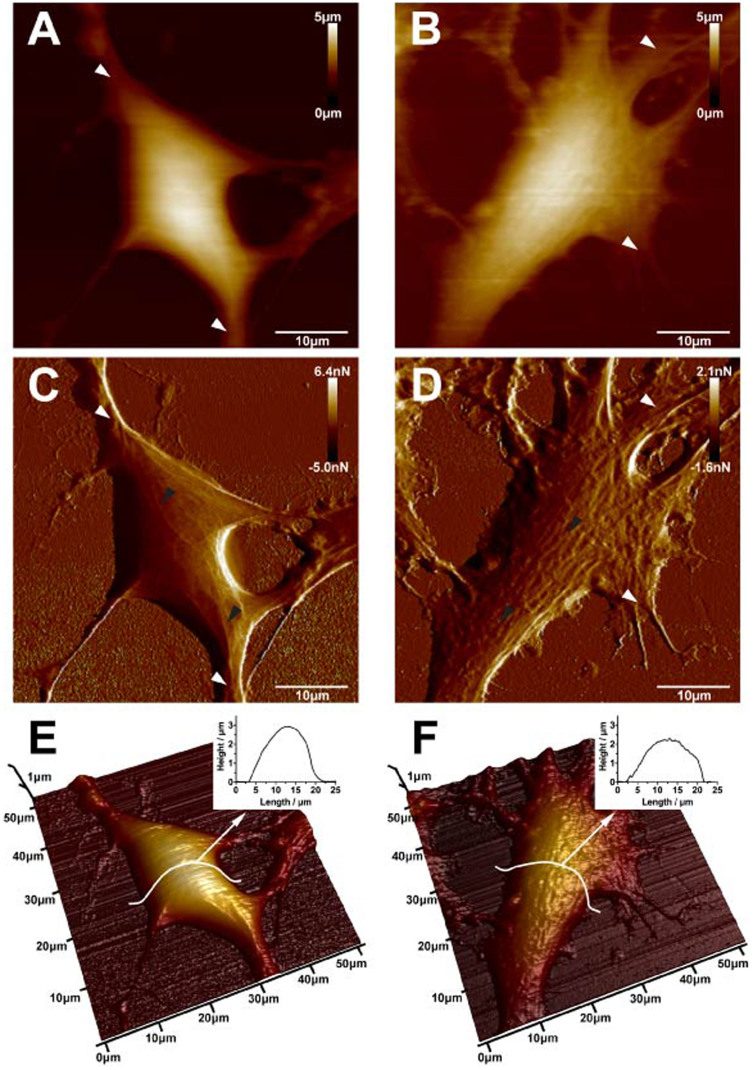
AFM topographic images of normal SH-SY5Y cells (A, C, and E) and 200 μM NMDA treated SH-SY5Y cells (B, D and F). (A) AFM height image, (C) peak force error image and (E) 3-D height image of normal neuronal cells. White arrows in (A) and (C) indicate neurite extension around the cell and black arrows in (C) indicate the filament structures. (B) AFM height image, (D) peak force error image and (F) 3-D height image of NMDA treated neuronal cells. White arrows in (B) and (D) indicate the neurite extension around the cell and black arrows in (D) indicate protuberances appeared and aggregated in the soma. (scale bar: 10 µm).

To study the morphogenesis of SH-SY5Y cells under stimulation of NMDA receptors, AFM images and surface roughness of normal and NMDA treated cells were obtained and compared, respectively. In addition to the standard AFM height images, the peak force error images were also acquired to display the finer details of the cell structure. A more clear and distinct neurite extension was found around the normal SH-SY5Y neuronal cell (indicated by white arrows in Fig. 3A, C). However, an obvious difference in the surface and neurites of the SH-SY5Y neuronal cell after 200 μM NMDA treatment for 1h was observed and the neurites were loosely distributed around the cell (indicated by white arrows in Fig. 3B, D). As particularly displayed in the peak force error images, the surface of the normal SH-SY5Y neuronal cell was smooth, and highly organized fine filaments appeared in the soma and neurite (indicated by black arrows in Fig. 3C). As compared with the normal neuronal cell, the surface of the NMDA treated neuronal cell was relatively rough and many protuberances appeared and aggregated in the soma (indicated by black arrows, Fig. 3D). The changes in surface morphology and filamentous structures after NMDA treatment were further observed in 3-D height images. As compared with the normal SH-SY5Y neuronal cell (Fig. 3E), the degraded neurite, rough surface and depleted fine filamentous structures were observed in the NMDA treated neuronal cell (Fig. 3F). In addition, a 33% reduction in cell height was observed after the NMDA treatment as indicated the 3-D height images of the normal (Figure 3, E inset) and NMDA treated neuronal cell (Figure 3, F inset).

**Figure 4:**
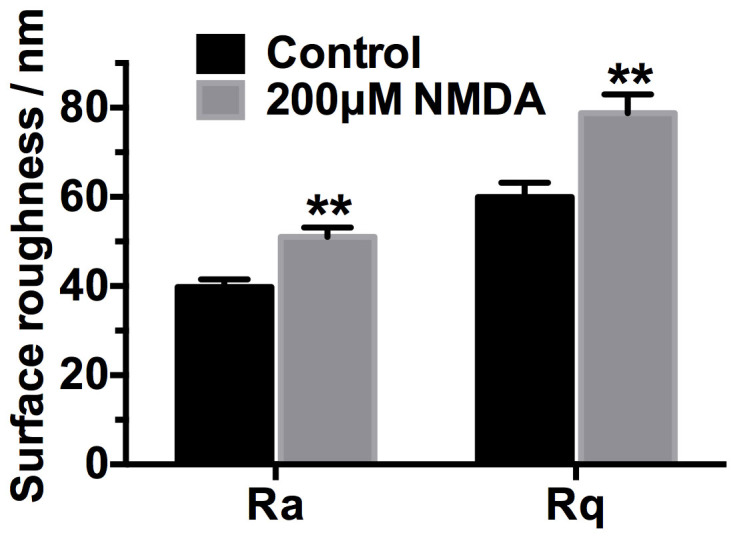
Surface roughness analysis of normal and 200 μM NMDA treated SH-SY5Y cells (mean ± SEM, ** p<0.01, Student's t-test). R_a_ is the average roughness and R_q_ is the root-mean-squared roughness.

To further quantify the morphological difference between the normal and NMDA treated neuronal cells, surface roughness values of SH-SY5Y cells were calculated by selecting a local region of 5 μm × 5 μm from the AFM height images. The average roughness (*R_a_*) and root-mean-squared roughness (*R_q_*) of the NMDA treated SH-SY5Y cells were increased by 28% and 31%, respectively (Fig. 4), which was consistent with the observation in the AFM images (Fig. 3C, D). The changes in morphology and surface roughness of SH-SY5Y cells detected by AFM were mainly caused by the subsequent reorganization of fine filaments after activation of NMDA receptors.

### Studies of the mechanical property of live SH-SY5Y cells after activation of NMDA receptors by AFM based nanoindentation

**Figure 5:**
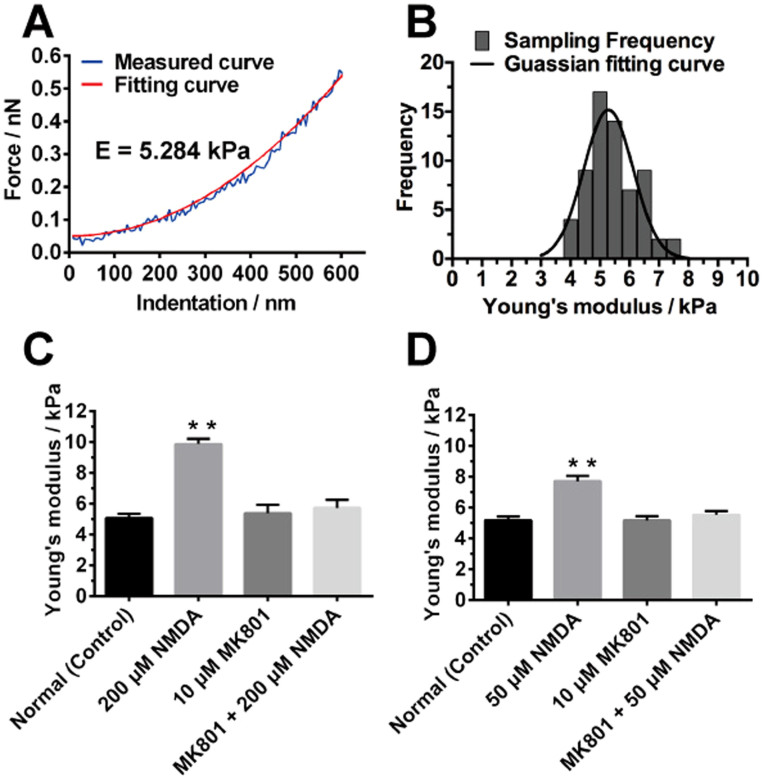
(A) Force-indentation curve of representative normal SH-SY5Y cells. The fitting curve was obtained by fitting the measured curve using the Sneddon model. (B) Frequency distribution of Young's modulus of representative normal SH-SY5Y cells. The solid curve was fitted by Gaussian distribution. (C) Statistical comparison of Young's modulus of SH-SY5Y cells under different conditions: without treatment (control); with 200 μM NMDA treatment for 1 h; with 10 μM MK801 treatment for 1 h; with 200 μM NMDA and 10 μM MK801 treatment for 1 h. (D) Statistical comparison of Young's modulus of SH-SY5Y cells under different conditions: without treatment (control); with 50 μM NMDA treatment for 1 h; with 10 μM MK801 treatment for 1 h; with 50 μM NMDA and 10 μM MK801 treatment for 1 h. Statistical analysis was conducted by SPSS using One-way ANOVA followed by Newman-Keuls post hoc test (**p<0.01). Data are represented as mean ± SEM (30 samples in triplicates for each condition).

Based on the results of the AFM imaging and surface roughness and the correlation between the cytoskeleton and mechanical properties of the cells that was previously observed in other reports^33^,^50^, we further examined the mechanical behavior of live SH-SY5Y neuronal cell under stimulus of NMDA receptors. AFM based nanoindentation was performed by probing the center of the SH-SY5Y neuronal cell. The indentation of the normal SH-SY5Y neuronal cell under the loading force of the AFM tip was calculated and the corresponding force-indentation curve was obtained. The curve was fitted using the Sneddon model and the Young's modulus of the normal SH-SY5Y neuronal cell was then calculated (Fig. 5A). The histograms of the measured Young's modulus from 64 force-indentation curves were plotted and the average Young's modulus of the normal SH-SY5Y neuronal cell was obtained by fitting the Gaussian distribution as shown in Fig. 5B. The experiment was repeated on the SH-SY5Y neuronal cell after 200 μM NMDA treatment for 1h and the corresponding average Young's modulus was shown in Fig. 5C. The average Young's modulus of the normal SH-SY5Y neuronal cell was increased from 5 kPa to 10 kPa after the NMDA treatment (Fig. 5C), which implied that the NMDA-treated neuronal cell was stiffer than the normal neuronal cell. In addition, MK801 (dizocilpine), a noncompetitive NMDA receptors antagonist, was used to block NMDA receptors. Since MK801 is an inhibitor of NMDA receptors, the MK801 could target NMDA receptors and reside in the transmembrane structures of NMDA receptors^51^. Consequently, the ion flow (e.g. Ca^2+^ influx) through NMDA receptors was blocked after treatment with MK801. In the present study, MK801 was used to inhibit the Ca^2+^ influx through NMDA receptors^52^. MK801 was added to the SH-SY5Y neuronal cell and the corresponding Young's modulus was obtained and compared with the normal and NMDA-treated neuronal cells. The result indicated that after adding 10 μM MK801 for 1 h, it had no significant effect on the Young's modulus of the SH-SY5Y neuronal cell (Fig. 5C). After adding the MK801 followed by NMDA on the SH-SY5Y neuronal cell, the Young's modulus value remained unchanged, which further showed the inhibitory effect of MK801 on the NMDA receptors (Fig. 5C). It also verified that the significant mechanical changes of SH-SY5Y cell were caused by the activation of NMDA receptors by NMDA and the subsequent Ca^2+^ influx. To further verify the effect of NMDA treatment on the neuronal cell, the concentration of NMDA was varied and set as 50 μM (Fig. 5D). Again, the Young's modulus of the SH-SY5Y neuronal cell was higher than that obtained from the normal neuronal cell and the neuronal cell treated with 10 μM MK801 (Fig. 5D). Furthermore, the Young's modulus was increased from 5 kPa to 8 kPa after 50 μM NMDA treatment, which was lower than the changes induced by the 200 μM NMDA treatment. It indicated that the increase in Young's modulus was affected by the concentration of NMDA. The higher concentration of NDMA could cause more Ca^2+^ influx to the cell, and thus disturb the actin filaments more intensively. Therefore, higher concentration of NMDA produced stronger effect on the morphological and mechanical properties of the SH-SY5Y cell.

### Time-dependent response of SH-SY5Y cells to the NMDA receptors activation

**Figure 6:**
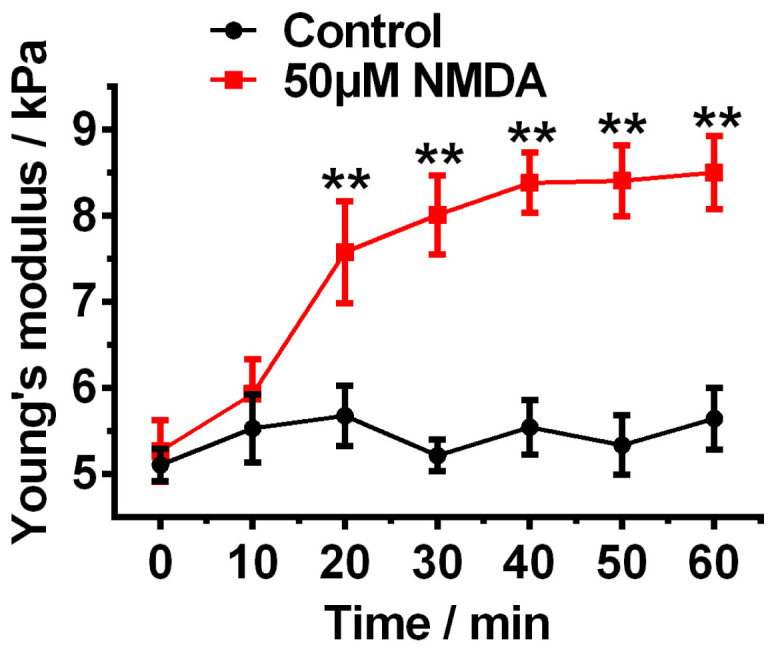
Experimental curve of Young's modulus of SH-SY5Y cells versus duration of the 50 μM NMDA treatment and statistical comparison with the curve of Young's modulus of normal SH-SY5Y cells over the test period (60 minutes). Statistical analysis was conducted by SPSS using One-way ANOVA followed by Newman-Keuls post hoc test (**p<0.01). Data are represented as mean ± SEM.

NMDA receptors are ion channels on the cytomembrane that mediate the transport of K^+^, Na^+^ and Ca^2+^, and the effect of NMDA receptors depends on the time of the NMDA receptors channel opening. Therefore, it is crucial to study the time course of the NMDA-receptors medicated neurodenegation. It is relatively difficult to apply conventional biological methods for monitoring the real time response of live neuronal cells. Here, AFM was employed to monitor the mechanical property and morphology of the neuronal cell over time. The Young's modulus of the SH-SY5Y neuronal cell before NMDA treatment was first recorded (the data as shown at 0 min in Fig. 6). After adding 50 μM NMDA to the SH-SY5Y neuronal cell, the Young's modulus was taken in 10 minutes time interval. The Young's modulus of SH-SY5Y neuronal cell was gradually increased with the time of the NMDA treatment, while the Young's modulus of the normal SH-SY5Y neuronal cell did not change significantly over the test period (Fig. 6).

**Figure 7:**
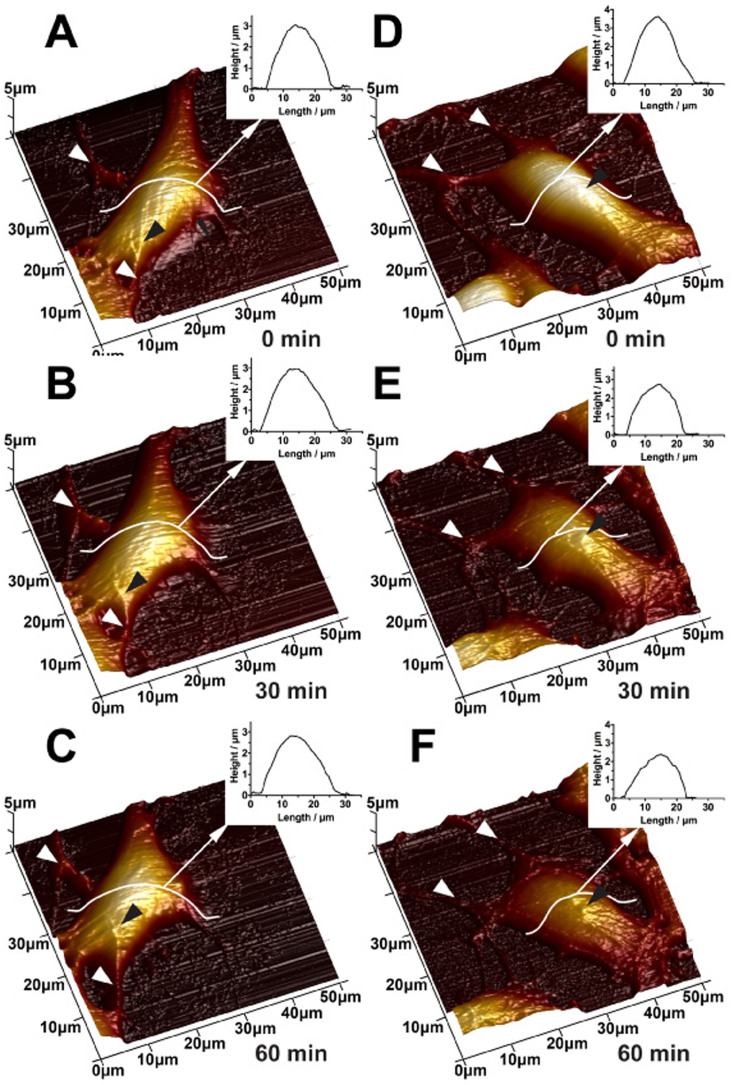
Time-course AFM imaging of normal (A–C) and 50 μM NMDA treated (D–F) SH-SY5Y cells. The AFM images were collected in 30 minutes interval. White arrows indicate neurite and black arrows indicate filament structures.

The time-dependent changes in surface morphology and filamentous structures of SH-SY5Y cells after NMDA treatment were also studied. AFM images of both normal SH-SY5Y neuronal cell and SH-SY5Y neuronal cell treated with 50 μM NMDA were obtained and taken in 30 minutes time interval. The images of normal SH-SY5Y cells (Fig. 7A, B, C) showed that obvious and distinct neurite extension (white arrows, Fig. 7A, B, C) and highly organized fine filaments appeared in the soma and neurite (black arrows, Fig. 7A, B, C). There was tiny reorganization in some parts of normal neuronal cells as observed in 3-D AFM height images over time, which might be caused by the normal action of live neuronal cells. As seen from the results obtained from the 50 μM NMDA treated neuronal cells, the cells exhibited similar morphology with normal neuronal cells before NMDA treatment (0 min) (Fig. 7D). After adding 50 μM NMDA for 30 minutes, changes in cell surface, neurite extension (white arrows, Fig. 7E) and filamentous structures (black arrows, Fig. 7E) were observed. After the NMDA treatment for 60 minutes, the cell was apparently changed including degradation of neurite (white arrows, Fig. 7F), depletion of filamentous structures (black arrows, Fig. 7F) and shrinkage of soma (cell height decreased from 3 μm to 2 μm).

The time-course AFM nanoindentation and imaging results demonstrate the cumulative effect of NMDA receptors activation. Together with the consistent morphological and mechanical property changes of the neuronal cell after adding NMDA for 1h, the real-time mechanical data collected by AFM can be used to reveal the progressive state of NMDA induced neurodegeneration.

### Identification of RhoA signaling pathway

**Figure 8:**
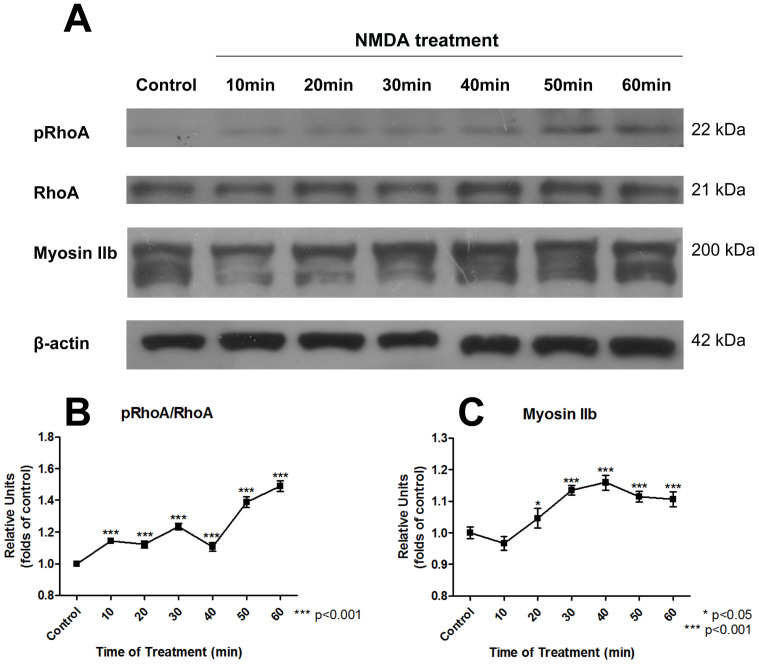
Western blot results showing the time-dependent response of RhoA and myosin IIb after 200 μM NMDA treatment. (A) Developed bands of pRhoA, RhoA, myosinIIb, and β-actin by immunoblotting. β-actin was used as a control. The immunoblotting of myosin IIb showed a form of multiple bands because of the existence of myosin IIb polymer. (B) Relative amount of RhoA (pRhoA/RhoA) was increased during NMDA treatment. (C) Myosin IIb was increased during NMDA treatment. Data are represented as mean ± SEM.

In order to further identify the downstream mechanism involving RhoA and myosin II under NMDA stimulus, Western Blot (WB) experiment was performed to investigate the effect of NMDA on RhoA and the downstream myosin II. The expressions of phosphorylated RhoA (pRhoA), RhoA, myosin IIb, and β-actin were measured by immunoblotting at different time points of the NMDA treatment as shown in Fig. 8A. β-actin was used as a loading control in the experiment. The relative amount of pRhoA/RhoA ratio and myosin IIb under the NMDA treatment was further measured. As shown in Fig. 8B and Fig. 8C, the amount of RhoA (pRhoA/RhoA ratio) and myosin IIb was significantly increased after a certain time period of the NMDA treatment. The increase of the pRhoA/RhoA ratio and myosin IIb further indicated that the treatment of NMDA activated the RhoA signaling pathway and induced the consequent activation of motor protein, myosin IIb, to mediate the neurodegenerative process. Eventually, the myosin II induces the increase in contractile forces among cytoskeleton. This results in the increase in pre-existing mechanical stress (prestress) in cytoskeleton, and based on the tensegrity model^27^, the cell becomes stiffer to resist the external loading.

## Discussion

Considerable effort has been devoted to discovering the pathogenesis and therapies behind neurodegenerative diseases such as Alzheimer's and Huntington's diseases, however, the effective diagnostic approach and treatment for neurodegenerative diseases are still under extensive investigation. Recent studies suggested that the NMDA receptor, a main glutamate receptors in CNS, plays a key role in mediating the synaptic plasticity of neuron that mainly modulates the learning and memory process^4^,^41^,^53^. The continuous and excessive stimulus of NMDA receptors was considered as one of the vital reasons to cause neurodegeneration^12^,^13^. However, the detailed mechanism of NMDA receptors-mediated neurodegeneration involved in different stages of the pathogenesis process is still largely unknown.

In the present study, we employed a nanotechnology approach based on the AFM platform to study the real-time degenerative behavior of live SH-SY5Y cells under stimulus of NMDA receptors. Here, we aim to collect the high-resolution topographic images and real-time mechanical properties under different stimulus conditions of NMDA receptors, so as to study the pathway involved in mediating the progressive phenotype of neurodegeneration in more detail.

NMDA receptors mediated Ca^2+^ influx to neurons and the subsequent cytoskeletal reorganization were well documented^10^,^54^,^55^. A large group of proteins, including the components and regulatory proteins of cytoskeleton, are found to be Ca^2+^ dependent^56^. Therefore, excessive stimulus of NMDA receptors leading to the accumulation of Ca^2+^ influx would possibly result in the disruption of the original dynamics of cytoskeleton. However, the principle of Ca^2+^ mediated cytoskeletal dynamics is poorly understood because of the difficulty in monitoring the cytoskeleton of live neurons in real-time. Compared with conventional biological methods, AFM was developed as a superior tool to monitor the cellular integrity (e.g., morphology and stiffness) and quantify the mechanical property of live cells in real time. By conducting the AFM imaging and nanoindentation, the changes in neuronal morphogenesis and mechanical properties were observed upon the NMDA receptors mediated neurodegeneration.

Our results indicated that the alternation in neuronal morphology was a key feature of the NMDA-receptors mediated neurodegeneration. AFM images revealed the fine and highly organized filamentous structures in normal SH-SY5Y cells. AFM was previously used for imaging the filamentous structures in other cells such as live fibroblasts^57^ and myoblasts^58^. It has also been reported previously that most of the actin filaments are concentrated in the cortex of neurons^26^. However, neuronal cells are the soft cell types as compared with other cells (10-fold softer than human keratinocytes^40^), the fine actin filaments of live neuronal cells are difficult to be observed by AFM imaging. To our best knowledge, we are the first to use AFM to observe the dynamic behavior of these fine filamentous structures in live SH-SY5Y cells. More importantly, after the neuronal cell exposed to NMDA for 1 h, the fine filaments in neuronal cortex were degraded and changed to some aggregated protuberances. The results were explained by the phenomenon that actin filaments in the cortex of neuronal cells are directly altered by excessive stimulation of NMDA receptors and regulation by Ca^2+^ influx^19^,^59^. In addition to the morphological characteristics, cell surface roughness was used as a quantitative indicator to reveal the effect of NMDA on the live neuronal cells. Cell surface roughness was previously used to reveal the phenotypes in tumor suppressor diseases^60^. The increase in surface roughness under stimulus of NMDA receptors is consistent with the rough surface observed in the AFM imaging, which further indicates the progressive degeneration of actin filaments in neuronal cortex. This result is also consistent with the previous study that excessive activation of NMDA receptors could lead to the depolymerization of actin filaments in the cortex of rat neurons^39^.

Furthermore, the changes in mechanical property of live neuronal cells observed in our AFM based nanoindentation experiments indicated that the NMDA induced cytoskeletal reorganization not only affect the cell topography, but also the cell mechanics. These changes can be further correlated to the disruption of RhoA signaling in neuronal cells under NMDA stimulation^18^. As a main regulator of actin filaments, it is well documented that Rho family GTPases played the major roles in controlling the neuronal plasticity in neuronal cells such as SH-SY5Y^61^,^62^,^63^. Upon completion of a series of regulation processes (e.g. stimulus of NMDA receptors^17^,^18^), myosin II motor proteins were activated by RhoA to generate contractile force among cytoskeleton and induce neurite retraction and soma shrinkage during the neurodegeneration process^14^. In the present study, the observed stiffening of SH-SY5Y cells under stimulus of NMDA receptors could be linked to the self-equilibrated and stable mechanical system of cytoskeleton based on the tensegrity model^27^. The stimulus of NMDA receptors induces the overall increase in contractile forces among cytoskeleton through the signaling pathway involving the activation of RhoA and myosin II motor proteins. This eventually results in the increase in pre-existing mechanical stress (prestress) in cytoskeleton, and thus the cell become stiffer to resist the external loading^27^. The results are also consistent with the observation in the increasing in modulus of elasticity due to the activation of NMDA receptors in cortical neurons^40^.

Neurodegenerative diseases are complicated and incurable because most of the current therapeutic approaches are mainly developed based on the symptomatic treatment or by slowing the progress of the disease pathology. Particularly, the clinical diagnosis is made based on the major neurodegenerative symptoms of the diseases, such as dementia and cognitive disturbances^64^. Therefore, there is an urgent need in developing a new approach for early diagnosis and prevention of neurodegenerative diseases. It is very difficult to study the real-time changes of the neuronal cell behavior under normal physiological or disease conditions with existing technologies. Based on the superior capability of AFM in observing live cells in real time, we performed AFM time-course experiments to monitor the immediate response of live SH-SY5Y cells under degeneration. Our data provided a direct correlation between the dynamic behavior of neuronal cells and the duration of NMDA treatment. The NMDA-mediated neurodegenerative process was further characterized by gradual structural and mechanical property changes of SH-SY5Y cells detected by AFM. Based on these significant results, the AFM based quantitative assessment can be applied to study other glutamate receptors that potentially induce neurodegeneration.

In conclusion, the findings presented here provide the direct link between the NMDA receptors-medicated neurodegenerative process and the mechanical behavior of live SH-SY5Y cells. We demonstrated that AFM can be used as an effective tool to observe the real-time gradual degeneration of SH-SY5Y cells under NMDA receptors activation, including degradation of neurites, depletion of filamentous structures and increase in cell surface roughness and stiffness. The present study can not only provide us more information to reveal the progressive neurodegenerative conditions involved in the pathological processes that cause neurodegeneration, but also provide an important diagnostic marker that is significant for screening anti-neurodegeneration drugs for the development of potential neuroprotective and therapeutic agent for neurodegenerative diseases.

## Methods

### Cell culture and drug delivery

SH-SY5Y human neuroblastoma cells (ATCC CRL-2266) were employed in the experiment and grown in the culture medium in a humidified incubator at 37°C with 5% CO_2_ flow for 7 days. Dulbecco's modified eagle medium containing nutrient mixture F-12 (Gibco, USA), 10% Fetal Bovine Serum (Gibco, USA) and 1% Penicillin-Streptomycin-Neomycin Antibiotic Mixture (Gibco, USA) was used as the culture medium. The cells were then subcultured by washing with 1 ml Phosphate Buffered Saline (PBS) (Gibco, USA) and treating with 1 ml of 0.25% trypsin-EDTA (Gibco, USA) for 2 minutes. For preparation of the cell samples for AFM measurements, the cells were seeded onto a glass microscope coverslip (WPI, MINI, 8 mm DIA) coated with Poly-D-lysine Hydrobromide (Sigma Aldrich) and cultured for 2 days before the AFM experiments. After two-day culturing, the cell samples were tested in a sealed petri dish with a self-prepared Mg^2+^ free medium. The medium was used in NMDA treatment experiments and included 145 mM NaCl, 5 mM KCl, 2 mM CaCl_2_, 20 mM HEPES and 5 mM glucose with PH 7.4^40^. The microscopy coverslip with cultured SH-SY5Y cells was then transferred to a petri dish with the Mg^2+^ free medium and placed on the AFM system under the controlled condition (37°C with CO_2_ inflow). The NMDA-treated sample was prepared by adding the NMDA (Sigma Aldrich, USA) in the Mg^2+^ free medium with cultured SH-SY5Y cells. For the single-time-point AFM experiment (1 h), the concentration of NMDA in the solution was 200 μM. As a comparison, a noncompetitive NMDA receptors antagonist MK801 (Sigma Aldrich) was used (concentration: 10 μM) and added to the Mg^2+^ free medium and 50 μM and 200 μM NMDA solution, respectively. For the time-course experiment, cell samples were treated with 50 μM NMDA and tested in different time intervals fixed at 10, 20, 30, 40, 50 and 60 minutes.

### AFM imaging and surface roughness analysis

High-resolution imaging, surface roughness and stiffness measurement were conducted by using a BioScope Catalyst AFM system (Bruker Nano, Santa Barbara, CA, USA). The system was equipped with a piezo-electric AFM scanner to drive the movement of the AFM probe in z-axis with the range of 16 μm. The maximum x-y scan range was 150 μm × 150 μm. An inverted optical microscope (Nikon ECLIPSE Ti, Nikon, Japan) was combined to the AFM system to locate the position of the sample and the AFM probe. An AFM probe (SCANASYST-FLUID, Bruker-nano, Santa Barbara, CA) with a spring constant of 0.7 N/m was used and the actual value was calibrated by a thermal tune method before each measurement. A new AFM scanning mode based on Quantitative Nanomechanical Mapping (QNM) and Peak Force QNM (Large Amplitude) in fluid (with amplitude of 1 μm and frequency of 0.5 kHz) was adopted to avoid damaging the cells during the scanning. During the AFM scanning, the maximum loading force was set at 4 nN and the scan rate was set at 0.3 Hz to obtain a moderate deformation of soma. During the AFM imaging, the scan size was set as 50 μm × 50 μm and the resolution was selected as 256 × 256, i.e. 256 lines along the vertical direction and 256 sampling points in each line).

The morphological difference between normal and treated cells was indicated by high-resolution AFM images and further quantified by analyzing cellular surface roughness (8 samples in each group). To acquire the representative surface roughness of cells, a local area of 5 μm × 5 μm was selected at the center of the cell. The average roughness (*R_a_*) and root-mean-squared roughness (*R_q_*) were subsequently calculated using equation (1) and (2), respectively^65^. 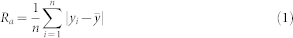

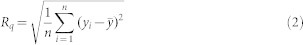
where *i* is the sampling order, *y_i_* is the *i^th^* value and 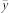 is the mean value of the height of *n* samples.

### Nanoindentation and stiffness analysis

The real-time monitoring of mechanical properties was conducted by AFM based nanoindentation. In this process, the AFM tip was employed as a nano-indenter to deform the cell to a specific depth. The displacement (*Z*) of the AFM tip in z-axis and the deflection (*d*) of the AFM cantilever were recorded. The loading force (*F*) was calculated according to Hooke' law by multiplying the spring constant (*k*) by the deflection of the AFM tip as shown in equation (3). The cellular deformation, indentation depth (*δ*), was calculated by subtracting deflection from the displacement of the AFM tip as shown in equation (4). The collected force-indentation curve was further fitted using the Sneddon model - a modified Hertz model for the conical shaped indenter^66^ and the Young modulus was calculated using equation (5). 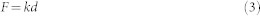

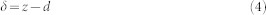

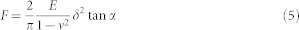
where *F* is the loading force calculated by equation (3), *E* is Young's modulus of the cell, *ν* is Poisson's ratio that is set to 0.5 for cell samples, *δ* is the indentation depth calculated by equation (4), and *α* is the half-open angle of the AFM tip that is set at 15°.

In the AFM indentation experiment, a soft silicon nitride AFM tip (SNL-10, Bruker-nano, Santa Barbara, CA) with a spring constant of 0.06 N/m was applied. The AFM tip was controlled to move to the center of the cell and the force curve was then taken. An array of multiple points was selected near the center of the cells and 64 force curves were finally taken for further analysis. For each force curve, 512 sampling points were collected at the frequency of 1 Hz. The acquired force curves were further fitted using the Sneddon model and the mean value of Young's modulus was obtained. 30 cells were tested to obtain the statistical value of Young's modulus for each condition.

### Western Blot Assay

After the NMDA treatment, the SH-SY5Y cells were washed with PBS for three times followed by protein extraction with lysis buffer. Equivalent amounts of samples (50 µg/well) were applied onto a 10% SDS-Polyacrilamide gel. After SDS-PAGE, the proteins were electroblotted to PVDF membranes (Bio-Rad) and blocked with 5% non-fat skim milk overnight. To analyze the expressions of different target proteins, the membranes were subsequently incubated in the primary antibodies supplemented with 2% Bovine Serum Albumin (Sigma). After washing with Tris-Buffered Saline and Tween 20 (TBST), the respective horseradish peroxidase (HRP)-conjugated secondary antibodies supplemented with 2% non-fat skim milk were applied onto the membrane at room temperature. The bands on the membranes were then visualized by Westsave Up (Abfrontier, South Korea). The immunoblotting of myosine IIb showed a form of multiple bands because of the existence of myosin IIb polymer. The quantification of myosine IIb was measured from the major band (200 kDa).

### Data analysis

AFM images and force curves were processed by the AFM offline software, NanoScope Analysis. Statistical data analysis was conducted by using Student's t-test for two groups and one-way ANOVA with post-hoc Newman-Keuls test for comparison of more than two groups. Data were presented as format of mean ± SEM.

## Author Contributions

Y.Q.F., C.Y.Y.I., C.N.P.L., Y.Z. performed the experiments. Y.Q.F. and K.W.C.L. analyzed the data. C.K.M.F., H.W.L., N.X., K.K.L.Y. and K.W.C.L. contributed the ideas and designed the experiments. All authors discussed the results and reviewed the manuscript.
